# Simulation and symbolic thinking in equations representing change

**DOI:** 10.1371/journal.pone.0339004

**Published:** 2025-12-18

**Authors:** Ambar Narwal, Emily R. Fyfe, Benjamin Motz

**Affiliations:** 1 Department of Psychological and Brain Sciences, Indiana University, Bloomington, Indiana, United States of America; 2 Cognitive Science Program, Indiana University, Bloomington, Indiana, United States of America; Public Library of Science, UNITED KINGDOM OF GREAT BRITAIN AND NORTHERN IRELAND

## Abstract

Learning simulations can enhance conceptual understanding in numerous education contexts, like physics, chemistry, ecology, and more. This conceptual understanding is often expressed symbolically using equations, and students are assessed on their ability to accurately express their ideas using these equations. In the current study, we explore how simulations can facilitate symbolic thinking, and specifically, how the presentation format of a simulation affects symbolic thinking in physics. Sixty-one undergraduate participants were assigned to one of three conditions: Animated Simulation, Textual Simulation and Control. In both simulation conditions, participants completed a set of tasks by manipulating values of key variables and observing changes in situations. These variables were speed, acceleration and time, and they influenced the motion of a ball in one dimension. While the Animated condition used animation of a moving ball to reflect these changes, the Textual condition reflected these changes through a structured text. No simulation was present in the Control condition. Participants then identified the equation which best described the situation. Compared to the Animated and Control conditions, participants in the Textual condition were more likely to identify the correct general form of the equation, where change was expressed through an addition term. These results highlight the importance of Textual simulations for equations describing one dimensional motion in physics.

## Introduction

The ability to use equations to solve problems is key to developing expertise in physics. This is why researchers and practitioners often consider this ability to be a relevant instructional target for physics education [1–3]. The solution to a physics problem begins with a conceptual understanding of the problem situation. This is followed by the selection of a relevant mathematical equation describing the relationships between key terms in the problem situation, which is then used to generate a solution. Early research on physics problem solving examined how students retrieve and apply physics equations from memory. For example, Chi et al. [4] theorized that cues in the environment activate certain principle-based schemas – structured networks of knowledge that connect problem situations to specific equations and strategies. Further, expertise in physics was equated with categorizing problems and activating schemas relevant to key physics principles (e.g., conservation laws) as opposed to superficial features (e.g., inclined planes or pulleys).

More recent work has expanded upon this view by suggesting that conceptual understanding not only enables selection of the appropriate mathematical equation, it also involves insight into how the equations’ symbols map onto the problem situation [5]. A successful student should not merely retrieve the correct equation from memory – a student should be able to “engineer symbolic relationships which express the verbal or graphical information needed to make progress in a problem” [6, p.31]. This has motivated researchers to study how students integrate their conceptual knowledge with symbols while solving problems in physics. To explain the role of conceptual knowledge during symbolic thinking, Sherin [7] proposed a set of knowledge elements called symbolic forms. Each symbolic form combines the idea to be expressed in an equation (e.g., starting point plus change) with a symbolic template (e.g., • ± ∆). The goal of the current study is to investigate how varying the presentation format of learning simulations influences the activation of these symbolic forms.

The presentation format of learning simulations can vary in multiple ways, and this variation has been shown to affect conceptual understanding. As mentioned above, Chi et al. [4] found that physics novices were sensitive to the literal features of problem situations, and that these concrete superficial details (rather than the underlying principles) influenced their learning performance. Indeed, research in cognitive psychology has demonstrated that varying learning materials on a spectrum of concreteness can impact learning from simulations [8,9]. A concrete presentation format emphasizes rich perceptual features detailing the objects and events we experience in the world. This similarity with our everyday experiences supports easy comprehension. In comparison, abstract materials are less realistic and only emphasize features which are critical for the concept to be learned. Even though abstract materials are less familiar, they promote generalization of knowledge to new situations by distilling the key essence of a phenomenon.

This concrete-abstract distinction exists not just in how learning materials are presented, but also how they are represented and processed in our minds. According to dual-coding theory, there are two information processing systems, verbal and non-verbal, underlying human cognition [10]. While the verbal system specializes in more abstract, language-like processing, the non-verbal (hereafter called visual) system is responsible for processing more concrete information. Even though referential connections exist between these two systems, information is dominantly processed in one system with activation later spreading into the other system.

In the educational context, dual coding theory has led researchers to examine how information presented in verbal and visual formats influences learners’ comprehension, retention, and transfer [11,12]. However, little to no work has tested how verbal and visual formats influence the mapping of concepts to symbolic forms in physics equations. The current study addresses this gap by comparing how verbal and visual presentation formats in learning simulations influence symbolic thinking in physics. In doing so, this study aims to clarify how different presentation formats support the conceptual-symbolic integration that underlies expert problem solving in physics.

### Tradeoffs between learning materials

Tradeoffs between concrete (visual) and abstract (verbal/symbolic) learning materials have been studied across many disciplines. Fyfe and Nathan [13] define concreteness in reference to idealization. An idealized format is a depiction which requires the least effort for a student to infer the invariant relation across situations and contexts. A less idealized format is more concrete and includes perceptual features and details that are irrelevant to the invariant relation in discussion. Presenting the concept of “two” using two red apples would be an example of a less idealized format because it includes surface features that are not needed to convey the core concept (e.g., red color, fruit category). In comparison, when we use the more abstract representation of the written numeral “2”, we have stripped away perceptual features of the apple, and represented the underlying concept in a completely symbolic, decontextualized way. The tradeoff between these learning formats arises, in part, because of the learner’s familiarity with these materials. Familiarity activates real-world knowledge which aids comprehension [14]. However, at times, increased familiarity hinders the novice learner from generalizing knowledge to different contexts.

There are numerous ways that learning materials can vary in their concreteness. For example, one could manipulate whether the learning materials are three dimensional or two dimensional (e.g., Fuson & Briars [15]) or whether the materials are presented as stories or with written symbols (e.g., Koedinger & Nathan [16]). In our study, we focus on the visual presentation format for an introductory physics problem, regarding the motion of a ball, presented to participants who have minimal prior training in physics. In this kind of physics scenario, one way of presenting the problem is through the use of iconic materials, such as a black circle (representing a ball) on a straight line (representing a frictionless plane). Often in physics simulations and animations, physical bodies are presented iconically, with graphics analogous to the events they denote. Iconic formats facilitate problem comprehension by activating knowledge elements attained through visual experience of the world, while avoiding overtly realistic elements which would be unnecessary for understanding the problem situation. This helps condense relevant concepts and highlights only the core features of the phenomenon of interest [17].

But while iconic materials provide a modestly idealized format, they are still concrete in the sense that learners are shown a visualization of the situation, communicating concepts via the visual (non-verbal) channel according to dual-coding theory. Alternatively, the results of a physics simulation might be presented in text, stripping all visual features, and describing the situation with words alone, thus leveraging the verbal channel. Text presentations have the benefit of facilitating comprehension by presenting students with a more precise lexicon [18]. Further, verbal descriptions can highlight the key structure of a visual representation [12,19], and research has suggested that verbal presentation formats have advantages over diagrammatic formats in some physics learning contexts [20].

It is not yet known how these tradeoffs between visual and verbal learning materials influence symbolic thinking in physics. On the one hand, our prior experiences with the physical world directly influence our beliefs about key concepts in physics. Sometimes these beliefs conflict with the concepts of Newtonian mechanics [21], and misconceptions arising from these commonsense beliefs persist following physics instruction [22]. This means that concrete visual materials might elicit familiar, intuitive but inaccurate knowledge of physics and make it harder to learn physics concepts. However, abstract verbal materials might have disadvantages as well. Meaning-making in physics is different from meaning-making in mathematics [23]. While equations in mathematics emphasize abstract relationships, equations in physics represent meaning about physical situations. Equations in physics are constrained by physical concepts and involve mapping this conceptual understanding onto mathematical expressions. While discussing student behaviors involved in solving word problems, Tuminaro and Redish [24] emphasized that this mapping process is a key struggle for novice physics learners. Given the importance of mapping meaning onto mathematical symbols, removing concrete materials completely might be detrimental to symbolic thinking in physics [25].

To summarize, both concrete and abstract learning materials can facilitate or hinder learning depending upon how they engage the visual and verbal systems, respectively. While the former can activate prior beliefs that do not align with formal principles in the discipline, the latter can reduce a problem situation to pure formalisms that describe only the abstract principles. While some theoretical frameworks argue that learning occurs best when multiple representational formats are used [13,26], theory should nevertheless be able to predict how different presentation formats enable learners to map concepts to formal symbols. In the current study, we compare these presentation formats separately in a physics simulation. One of the conditions uses visual animations of a moving ball to illustrate the outcome of the simulation. The other condition, which is more abstract, communicated the ball’s motion to the learner in a textual format. Even though dual coding theory predicts that learners might have the best performance on memory based tasks [11] when these two presentation formats are combined, the current study is instead interested to understand which presentation format more directly supports symbolic thinking, specifically, during a physics learning simulation.

### Learning simulations

Researchers have investigated the role of simulations in a variety of educational contexts. In comparison to static learning materials, simulations incorporate dynamism through animations and interactivity through controllable parameters [27, p. 289]. Animation here is defined as “the process of generating a series of frames containing an object or objects so that each frame appears as an alteration of the previous frame in order to show motion” [28, p. 132]. Berney & Bétrancourt [29] conducted a meta-analysis of studies which examined the role of animation in learning. Studies included in this analysis varied across four key factors: instructional materials (iconic/abstract), modality of the information accompanying animation (visual text/narration/no information), instructional domain and learning outcomes. Overall, the authors found a small but significant positive effect of learning with animations in comparison with static graphics. This effect was significant for animated visualizations that used iconic learning formats. While an auditory narration with the animation was better than visually presented text, animations were most effective when there was no accompanying textual information (p.27). This finding runs contrary to existing theoretical accounts which argue for the use of multiple representations. It’s possible that for some learning contexts, the individual benefits of different representational formats may not be cumulative when combined.

Within the domain of physics, researchers have found that human judgments are aligned with the principles of Newtonian Physics when situations are presented through animations [30]. In addition to animation, the interactive component in simulations allows learners to exercise parametric control over relevant variables and experience different states of the situation [31]. By simulating behaviors that align with formal physics principles, simulations in physics can update existing beliefs about a mechanism. Prediction is another way in which learners can update their existing beliefs. In the context of mechanical systems, Hegarty et al. [32] found that asking students to predict the behavior of a system improved learning about the underlying mechanism of a system. This was because such questions activated prior knowledge and helped learners identify knowledge gaps in understanding [33]. In our study, both experimental conditions incorporated this prediction component through goal-driven simulations.

Given that simulations have strong potential to facilitate learning, a common instructional resource is the Physics Education Technology (PhET) simulations [34]. Depending upon the phenomenon under observation, PhET simulations allow learners to vary values of key variables and observe changes to the system. Banda and Nzabahimana [35] recently reviewed studies investigating the effect of PhET simulations in conceptual understanding in physics. Numerous studies [36–39] documented improvements in conceptual understanding as a result of using PhET simulations.

Academic literature on simulations often compares visual learning materials in a static versus dynamic format. In such studies, verbal information plays a secondary role – often by directing attention to parts of the simulation which are otherwise not salient [40]. For example, Mayer and Anderson [12] found that animations (explaining mechanical systems like pumps and brakes) led to better problem-solving outcomes when they were supplemented with verbal narrations. Thus, textual materials (e.g., verbal narrations, written descriptions) have mostly been investigated in addition to primary visual learning materials. However, it is possible that when presented in an interactive goal-driven context, textual materials could update existing beliefs of a system similar to animated materials. From a dual coding standpoint, this question is crucial because animated simulations primarily engage the visual system, while textual simulations engage the verbal system. To examine how both these simulations facilitate symbolic thinking, we explore this contrast through Animated and Textual Simulation conditions in our study.

### Current study

The current study examined how the presentation format of materials used in learning simulations influences symbolic thinking in physics. The target equation for the study was *q = p + rt*, which describes the final speed (*q*) of an object in terms of its initial speed (*p*) and acceleration (*r*) over a specified period of time (*t*). The variables were intentionally relabeled (e.g., q, p, r, t instead of v, u, a, t) to minimize participants’ reliance on previously learned physics formulas. The choice of this equation was motivated by two factors. First, we wanted to investigate the influence of different presentation formats in novice physics learners. This necessitated the choice of a simpler equation with a limited number of variables. We initially piloted the study with another equation that had a multiplicative form, but that task was too difficult for physics novices (zero participants were able to correctly identify the multiplicative form). Second, conceptually, the target equation of motion is important for kinematics, and subsequent mechanics often builds upon this knowledge. Learning this equation requires mapping the conceptual knowledge of acceleration, initial speed, and time to their respective symbols to determine a moving body’s final speed.

Both the Animated Simulation condition and Textual Simulation condition were aimed at facilitating this mapping process from concepts to symbols. The interactive component in the simulations allowed participants to manipulate values of key variables and observe changes in the situation. The situation represented by the equation involved motion of a ball in one dimension. For the Animated condition, changes in the situation were illustrated through the animation of a moving ball. Participants could immediately perceive how changes in each variable influenced the ball’s motion. For example, increasing the initial speed (while keeping other variables constant) caused the ball to begin moving faster from the very first frame, whereas increasing acceleration led to a gradual increase in speed over time. This dynamic visual feedback could allow participants to perceive the distinct causal roles of the variables, specifically how initial speed determines starting motion and how acceleration governs change in speed. In comparison, for the Textual condition, changes in the situation were stated using textual descriptions. This description didn’t change with each frame, but was displayed once at the end. A third Control condition had no simulation or interactivity, and only gave participants textual descriptions of key variables in the context of a ball in motion.

**Fig 1:**
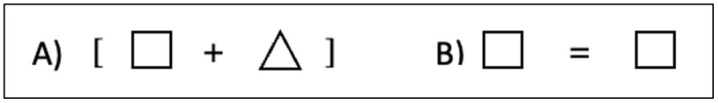
Symbolic Templates. This figure illustrates symbolic templates for **A)** Base + Change, and **B)** Balancing.

To evaluate a condition’s effectiveness in mapping conceptual knowledge gained from the problem situation onto symbols, we used an equation identification task. This task asked participants to choose the equation which best represented the problem situation from a list of six options. While there was only one correct option (*q = p + rt*), two other options included a symbolic form similar to the correct equation. As discussed in the introduction, a symbolic form is expressed as an amalgamation of two components, a conceptual schema and symbolic template. While the conceptual schema captures the idea to be expressed in an equation, the symbolic template is the syntactic component of the symbolic form. Fig 1 shows two examples.

The geometrical shapes represent kinds of variables. The variable in use can vary but the grammar of the final equation is constrained by the symbolic template. For example, Base + Change connects ideas involving a starting point (Base) and an update term (Change) with the addition operator. Our target equation closely resembles this form. In our equation, initial speed (*p*) is the base term and change in speed due to acceleration over time (*rt*) is the change term. Two other options (*(p + r)t* and *(p + r/t)*) also use the addition operator to connect the initial speed term (*p*) with the change that happened due to acceleration. This stands in contrast with the three remaining options that directly combine initial speed (*p*), acceleration (*r*) and time (*t*) through multiplication and division operators (other three options were *pr/t, prt, pt/r*; see Fig 3 for a full list of response options). Therefore, apart from the correct equation, we were interested in assessing whether participants chose options with the accurate symbolic form. Since the addition operator distinguishes this form from other options, we refer to it as the *addition* form going forward.

**Fig 2:**
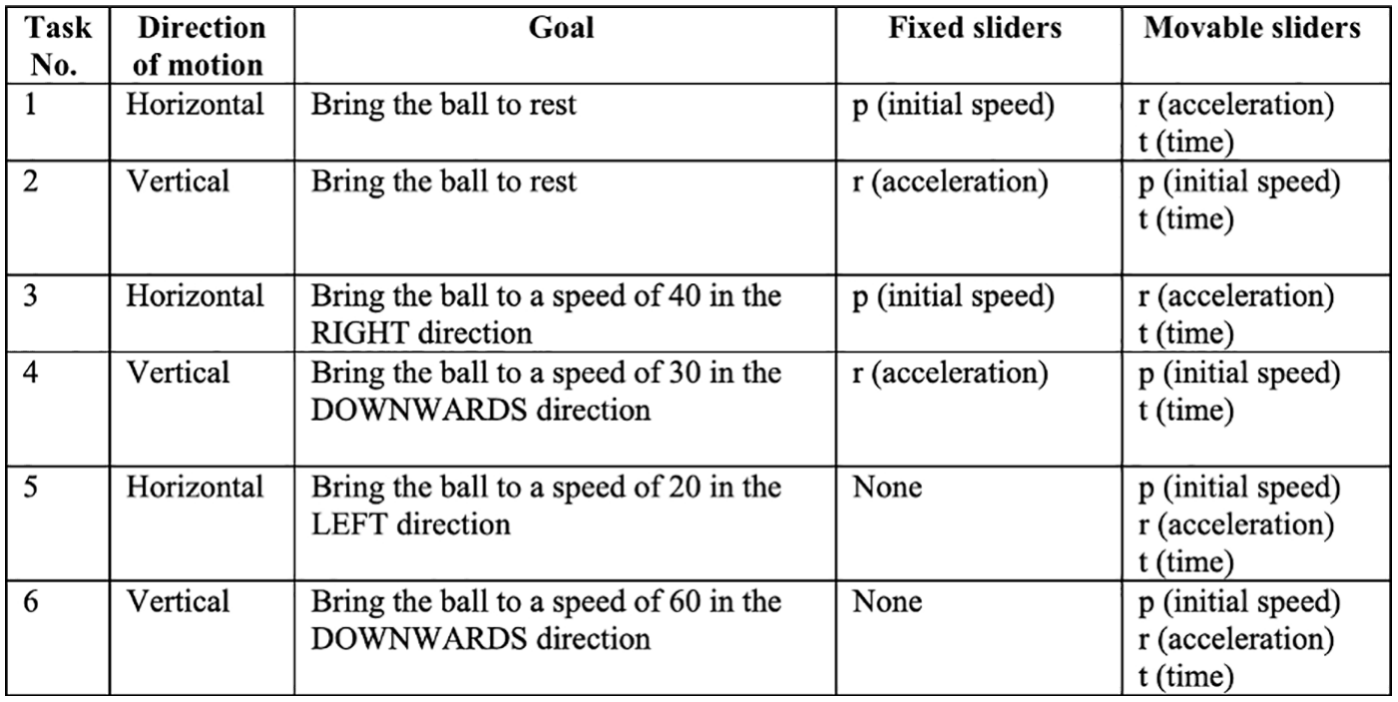
Factors that Varied Across Interactive Tasks. The first two horizontal and vertical goals had fixed initial speed (*p*) and acceleration (*r*) values respectively. The last two goals allowed participants to vary all three variables.

From the theoretical perspective of dual-coding, the textual simulation condition would primarily engage the verbal system while the animated simulation condition would engage the visual system. Students map the conceptual knowledge gained from engaging both these systems to the equation of motion. We hypothesize that compared to the animated simulation condition, the textual simulation condition would be better at forming associative links between the textual descriptions and mathematical symbols. This is because both text and symbols would be processed in a similar manner by the verbal system. The discrete and structured nature of text would facilitate better mapping to an equation which also consists of discrete symbols. In comparison, while animated simulation may strengthen an intuitive understanding of motion, its continuous, image-based format may not directly support symbolic mapping. Relative to the control condition, we expect that the interactive component in the other two conditions will facilitate mapping of relevant concepts to symbols, and thereby increase the likelihood of selecting the correct symbolic form and the objectively correct equation.

## Method

All the materials, de-identified data and scripts for reproducing analyses and figures are publicly available via the Open Science Framework (OSF; https://osf.io/3urjb/). The study was conducted under a research protocol approved by the Indiana University Institutional Review Board (IRB #17102), and all volunteers provided explicit consent to participate. The present study was exploratory in nature and was not preregistered.

### Participants

Sixty-one undergraduate psychology students from Indiana University participated in this experiment in exchange for partial course credit. All participants completed a single in-person experimental session with a trained researcher and provided written consent to participate and to have their session recorded. The participants were randomly assigned to one of these three conditions: Animated Simulation (*n* = 21), Textual Simulation (*n* = 20) and Control (*n* = 20). For analyses, the training simulation data was excluded for one participant in the Animated condition due to a technical error, but that participant’s response to the primary outcome measure (the equation identification task) was still evident in the video recording and analyzed accordingly. Based on self-report, half of the participants had never taken physics in the past, while 72% of participants had taken at least one semester of calculus. The gender distribution of these participants was 70.5% female and 29.5% male. With regards to their year of study, 75.4% were freshmen, 13.1% sophomores, 4.9% juniors and 6.5% seniors. Of all the participants, 81.9% reported themselves to be White, 9.8% Black, 3.3% Asian, 3.3% Hispanic and 1.6% belonged to a mixed race.

### Materials

The experiment was scripted in JavaScript and presented in a web browser. The materials included an initial assessment, the training simulations, an outcome measure, and a demographic questionnaire.

#### Initial assessment.

To collect background information, participants completed an initial questionnaire in which they answered objective questions pertaining to their highest level of education in math and physics. Additionally, since the equation in focus involved speed and acceleration, participants had to choose the correct definitions of speed and acceleration from given options and provide subjective responses for the units associated with these concepts.

#### Simulation.

Participants in the Animated and Textual Simulation conditions were first exposed to a scenario of a ball in motion. A two-dimensional image of a static ball was shown on a flat surface represented by a horizontal line. Alongside this image, participants were given descriptions of key variables essential to the balls’ motion. To ensure that participants did not have to memorize what the key variables represented, their definitions were displayed in the left corner of all subsequent screens.

*(1) Animated Simulation:* Once participants read the scenario, the next six screens had goal-based interactive tasks. These tasks involved manipulating values of initial speed (*p*), acceleration (*r*), and time (*t*) variables by dragging respective sliders. The goal was to get the ball to a certain speed in the left or right direction. Of the six interactive tasks, three involved motions of the ball in the horizontal direction and three involved motions in the vertical direction. Also, the initial speed (*p*) slider was fixed for the first two horizontal tasks and the acceleration (*r*) slider was fixed for the first two vertical tasks. These sliders were fixed initially to gradually increase the difficulty of the task. Details of each task in their order of presentation are shown in Fig 2.

**Fig 3:**
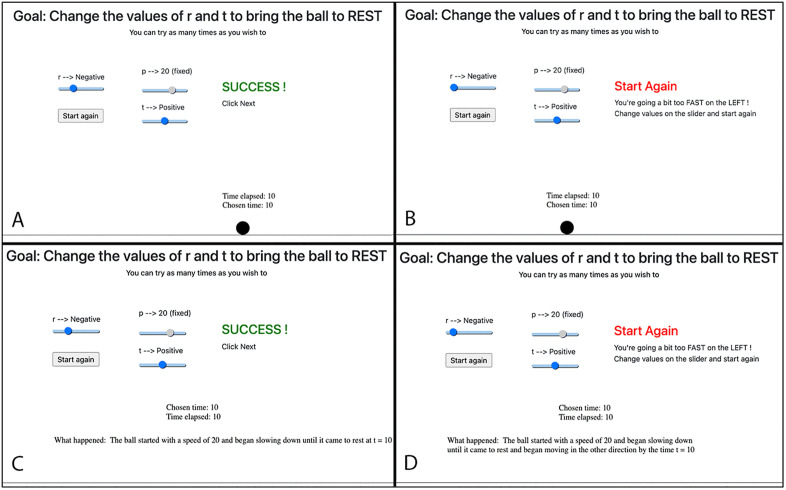
Comparison of different Outcome States in both Simulation Conditions. This figure illustrates **A)** Success state in Animated condition, **B)** Fail state in Animated condition, **C)** Success state in Textual condition, and **D)** Fail state in Textual condition. The moving ball is replaced by text in the Textual condition. r, p and t represent acceleration, initial speed and time respectively.

Once participants adjusted values on the sliders and clicked on the “Start” button, those in the Animated condition saw an animation of the ball moving. The motion was in accordance with the values of variables and lasted for time t. When the chosen time elapsed, two things happened. Participants got textual feedback saying “Success” or “Start Again”. *Success* meant that the ball had achieved the desired goal state. If the feedback said *Start Again*, there was additional information stating whether the ball was faster or slower than the desired speed (Fig 3). Participants could proceed to the next screen if they succeeded in the task, and they had to start again if they failed. After ten failed attempts, participants were given the option to proceed without succeeding in the task.

*(2) Textual Simulation:* Similar to the Animated condition, this condition also presented the same scenario and required participants to engage with the same six goal based interactive tasks. The direction of motion and the sliders that could be manipulated were also the same. However, after adjusting values on these sliders, once participants clicked “Start”, the time counter started. When the elapsed time equaled chosen time, the trajectory followed by the ball was explained through text. So rather than watching the ball move, they read this text:

*What happened: The ball started with a speed of 20 and kept speeding up until t =8*

Depending upon the changes in the ball’s speed and trajectory, the textual description varied. Sometimes, the ball just sped up and attained its goal. In other cases, it sped up, slowed down, changed direction and then attained the desired speed. For example, when a participant achieved the vertical goal of bringing the ball to a speed of 30 in the downwards direction, participants read this text:

*What happened: The ball started moving upward, kept slowing down, changed direction, until it came to the desired speed at t = 8*

To ensure that each distinct behavior of the ball was mapped appropriately to the text, there were a total of 33 textual descriptions across 6 goals. 16 out of these 33 possible textual descriptions never used the word “and” to separate the start of the ball from the change that happened due to acceleration. Alongside this text, the participants also got textual feedback saying “Success” or “Start Again” as in the Animated condition.

To summarize, everything was same across both conditions, except in the Textual condition, the participant had to infer the ball’s motion through text (see Fig 3).

*(3) Control:* In this condition, the six interactive tasks were replaced by answering six standard quantitative reasoning problems sourced from Graduate Record Examination (GRE) problem sets online. After the participants tried these GRE problems, they were exposed to the scenario of a ball in motion and descriptions of key variables essential to its motion were provided. However, participants in the control condition simply read this information but did not complete any activities related to the ball’s motion.

### Outcome measures

**Fig 4:**
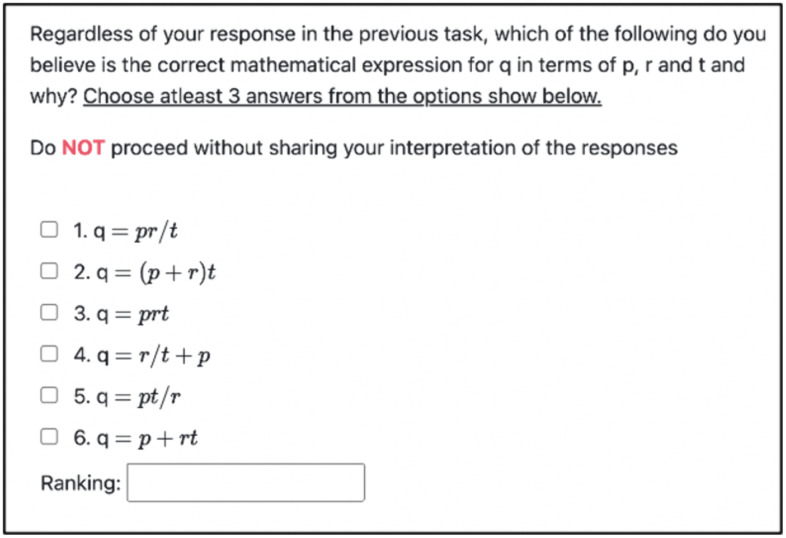
Speed Equation Identification Task. The figure shows six options that are presented to participants in the equation identification task. Option 6 is the correct equation while options 2, 4 and 6 use the addition form. r, p and t represent acceleration, initial speed and time respectively.

After the training activities, participants completed an equation identification task. Specifically, they were shown a series of mathematical expressions for the final speed variable q. Participants had to identify the three best options from the six options (Fig 4). They also had to rank these three options from best to worst. Three of these options (labeled 2,4,6 in Fig 4) used the addition form while the other three options (labeled 1,3,5 in Fig 4) did not.

#### Demographic questionnaire.

The final section collected information regarding participant’s age (in years and months), year in college, gender, race/ethnicity, current GPA and ACT/SAT scores.

### Procedure

Participants arrived at a scheduled time for an in-person session. They were seated at a comfortable workstation in a small office. After providing consent, participants completed the initial assessment questionnaire and then proceeded to the training activities (i.e., simulations in the Animated and Textual conditions and GRE problems in the control condition). The simulations were followed by the equation identification task. The experiment ended with participants entering relevant details in the demographic questionnaire. Apart from these items, participants also completed a few additional exploratory items that are not reported here. All the materials were presented virtually on a Macintosh iMac. On average, participants spent 50 minutes on the experiment.

Throughout the session, a researcher was seated in the testing room with the participant. The researcher resolved any queries the participant had and supplemented the protocol with verbal instructions when it was required. Additionally, on several occasions during the experiment, the researcher asked reflective questions and sought verbal explanations from the participant. These verbal explanations generated meaningful qualitative data and provided adequate context to the participant’s responses. The experimenter followed a scripted protocol which was consistent across conditions.

All the interactions between the researcher and participant were video recorded. Additional data was captured through a screen recording of the whole experiment. For the interactive tasks in the Animated and Textual conditions, the values of slider variables, final speed, goal status and attempt count were all collected. Similarly, responses on all assessments and questionnaires were recorded separately from the video and screen recordings.

## Results

### Equation identification

For the equation identification task, participants first selected three out of six equations and then rank ordered their list of three equations describing the relationship between initial speed (*p*), acceleration (*r*), time (*t*), and final speed (*q*). For the current analysis, we examined participant’s first choice in their ranking and scored it at two levels: (1) whether it was the correct equation (*q = p + rt*), and (2) whether it was any of the three equations that had the *addition* form (options 2,4,6 in Fig 4).

In terms of the correct choice, we found that only 16 out of 61 participants ranked the correct equation as their first choice (26.2%). Eight of these 16 participants were in the Textual condition, and the remaining eight participants were equally distributed between the Animated condition and the Control condition. We ran a logistic regression with Control condition as the referent. We found that the Textual condition did not significantly predict participants’ choice of the correct response (*p + rt*; *B* = 0.980, *SE* = 0.721, *p* = 0.174). Animation condition also did not significantly predict participants’ choice of the correct response (*B* = −0.060, *SE* = 0.788, *p* = 0.938).

**Fig 5:**
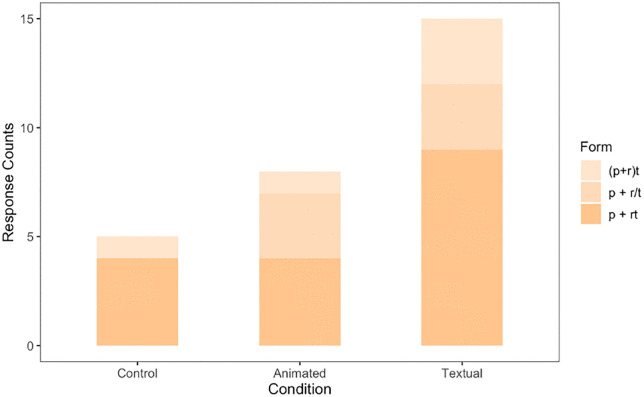
Addition Form Response Counts. This figure shows counts of addition form options across all three conditions. The addition form count is highest for the Textual simulation condition, followed by the Animated simulation condition and the Control condition.

**Fig 6:**
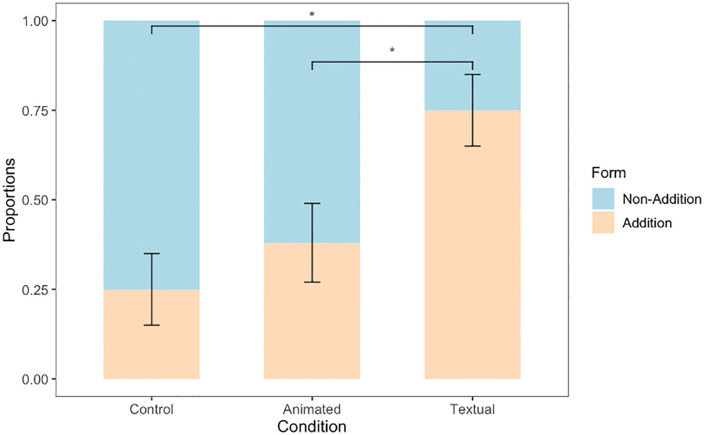
Equation Identification Response. This figure shows proportion of responses adhering to the Addition and Non-Addition form across all the three conditions. Proportion of addition form responses in the Textual condition was significantly higher than both the Animated and Control condition. Error bars represent standard errors. *p < 0.05.

In terms of selecting any equation with the *addition* form, we found that 28 out of 61 participants chose one of the options that included the *addition* form (45.9%). Fifteen of these 28 participants were in the Textual condition (Fig 5). To test whether this effect was significant, we ran a logistic regression with selection of the *addition* form as the outcome variable and condition as the predictor variable (with the Control condition as the referent). Regression coefficients indicated that compared to control, Textual condition (*B* = 2.197, *SE* = 0.730, *p* = 0.002) was a significant predictor of the choice of *addition* form. The Animated condition was not significantly different from the Control condition (*B* = 0.613, *SE* = 0.684, *p* = 0.370). To test if the Textual condition was significantly different from the Animated condition, we ran a supplemental logistic regression with Animated condition as the referent. Regression coefficients indicated that compared to the Animated condition, Textual condition (*B* = 1.584, *SE* = 0.684, *p* = 0.020) was a significantly better predictor of *addition* form (Fig 6).

To examine whether the observed difference in participants’ selection of the *addition* form was solely due to their randomly assigned condition, we calculated pairwise correlations between all prior knowledge variables and test conditions. For context, there were six of these prior knowledge variables and five of them did not differ across conditions. These five prior knowledge variables that did not differ across conditions were: (1) rating the number of physics classes taken compared to others; (2) defining speed; (3) defining acceleration; (4) reporting correct unit for speed; (5) reporting correct unit for acceleration. The sixth variable assessed their prior math experience, and it differed by condition. Thirty-six out of 61 participants (59%) reported taking the Same number of math classes as other students. From the rest of the participants, 19 (i.e., 31%) reported taking Slightly More or A Lot More math classes compared to other students. Of these 19 participants, 9 participants were in the Textual condition, 6 were in the Animated condition and 4 were in the Control condition. When compared across conditions, this prior math experience variable was significantly correlated (*r*(59) = 0.274, *p* = 0.032) with the Textual condition variable. Compared to other students their age, participants in the Textual condition reported taking more math classes.

We examined whether the effect of condition on selecting the addition form remained significant when taking prior math experience into account. To do so, we ran a logistic multiple regression with prior math experience as an additional predictor variable. The Textual Condition remained a significant predictor of the choice of *addition* form relative to the Control condition (*B* = 1.964, *SE* = 0.750, *p* = 0.008), and relative to the Animated condition, (*B* = 1.454, *SE* = 0.696, *p* = 0.036). Prior math experience (*B* = 0.681, *SE* = 0.515, *p* = 0.186) was not a significant predictor in both these cases.

### Simulation data

To better understand why the Textual condition facilitated participants’ choice of the *addition* form, we analysed behavioural data that was recorded during the simulation phase of the experiment. First, we examined the success rate of participants in the Animated and Textual conditions during the training activity. Each participant attempted 6 goals (e.g., get the ball to stop). On average, participants succeeded on 5.7 goals (95.4% overall success rate). The difference in the success rates between the Animated condition (96.7%) and the Textual condition (94.2%) was not significant (*t*(35.8) = 0.788, *p* = 0.435).

Second, we examined the effort it took participants to achieve success. We did so by calculating the number of attempts it took participants to get to the success state. Animated Condition participants took an average of 5.3 attempts (*SD* = 4.14) to achieve success on each trial, and Textual Condition participants took an average of 5 attempts (*SD* = 4.26) to get to a success state on each trial. The difference in means was not significant (*t*(226) = 0.463, *p* = 0.643).

Third, we examined if the use of the word “and” during Textual simulation trials influenced the choice of the addition form. Out of 621 trials in the Textual condition, 320 trials (51.5%) never used the word “and.” For each participant, we computed the proportion of trials that included “and” and used this measure as a predictor in the logistic regression model. Because this variable was only relevant to the Textual condition, the analysis was restricted to participants in that condition. Prior math knowledge was included as an additional predictor variable. We found that proportion of trials which used the word “and” was not a significant predictor of the choice of addition form (*B* = 3.813, *SE* = 5.622, *p* = 0.498).

Finally, we examined the different strategies participants used to get to a success state. For example, participants could adopt a strategy to only change one variable at a time, or alternatively, participants could adjust multiple variables during a single attempt. Past research finds that changing just one variable value at a time (i.e., the Control of Variables [COV] strategy) is foundational to scientific thinking [41] because it allows you to draw explicit causal links between the changed variable and the outcome variable.

We analysed if there was a difference in the use of this strategy across Animated and Textual conditions. If a participant only changed one variable value in successive attempts to reach the goal, the COV strategy variable was coded 1 for that attempt, and then, for each participant, we calculated the proportion of trials which used the COV strategy. We find that across both conditions, more than 70% of trials used the COV strategy. For participants assigned to the Animated Condition, 0.70 (*SE* = 0.02) proportion of trials on average used the COV strategy. In comparison, Textual Condition participants on average had 0.81 (*SE* = 0.03) proportion of trials which used the COV strategy. This difference in proportions between both conditions was statistically significant (*t*(32) = 2.798, *p* = 0.008), and this was true even after controlling for prior math experience (*B* = 0.118, *SE* = 0.037, *p* = 0.003).

To summarize, participants in the Textual condition were more inclined to use the COV strategy than participants in the Animated condition, and further, participants in the Textual condition were more likely to correctly select an *addition* form in the equation identification task. To determine whether the difference in use of the COV strategy predicts the difference in choice of *addition* form in both the Textual and Animated conditions, we ran a logistic regression with *addition* form as the outcome variable and proportion of COV strategy use as a predictor variable. We found that the COV strategy variable did not significantly predict the choice of *addition* form (*B* = −1.082, *SE* = 2.490, *p* = 0.664). This meant that those participants which used this strategy more often during simulations were not more likely to choose the *addition* form options in the equation identification task.

### Qualitative verbal responses

To better understand participants’ selections on the equation identification task, we qualitatively explored their verbal reports. After making and ranking their selections, we asked participants to share their thought process behind the rankings. Here, we provide some representative verbal responses that capture their understanding of the relationship between initial speed (*p)*, acceleration (*r)* and time variables (*t)*.

Our exploration of these verbal responses suggested that participants who chose one of the three equations in the non-addition form conceptualized initial speed (*p)* and acceleration (*r)* as variables which directly influenced each other. Table 1 presents some verbal quotes that illustrate this conceptualization.

**Table 1:** Explanations of participants who chose non-addition forms.

Quotes
“Just because the format of it makes most sense to me in the sense where I feel like initial speed and acceleration would have a direct impact to each other.”
“And because the acceleration, I mean your initial speed is affected by the acceleration and it’s all over time. And then some of them like just add them all together. It just doesn’t make any sense. It’s not going to, we’re not like something’s not being affected by something else just by adding it.”
“I use multiplication because it’s something that they (referring to initial speed and acceleration variables) interact with each other.”
“Initial speed and acceleration kind of work together and then over that span of time would get me my final speed.”
“I feel like initial speed and acceleration would have a direct impact to each other.”

While choosing the non-addition form meant choosing one of the three options (1, 3 or 5 as shown in Fig 4), the large majority (82%) of the participants who chose a non-additive form chose option 1 (*q = pr/t*). This option connected initial speed (*p)* and acceleration (*r)* using a multiplication operator.

In comparison, participants who chose one of the three equations in the *addition* form conceptualized initial speed (*p)* and acceleration (*r)* as variables which should be kept separate from each other. Table 2 presents some verbal quotes which illustrate this conceptualization.

**Table 2:** Explanations of participants who chose addition forms.

Quotes
“Initial speed is separated because it’s like our starting point... Because I feel like initial speed needs to be by itself.”
“So it makes more sense that they would be like singled off from the initial speed...I feel like initial speed wouldn’t be multiplied by time. Mm hm. Just because, like, it just doesn’t make sense because you have an initial speed like that’s separate from whatever you’re adding onto it.”
“Because it (initial speed variable) pretty much is separate as far as like the scenario goes because you’re not going to multiply it by your acceleration.”

In fact, of the 28 participants who chose the addition form, 12 (43%) suggested in their verbal explanations that initial speed (*p)* and acceleration (*r)* should be kept separate from each other. Only 3 (11%) participants suggested that initial speed (*p)* and acceleration (*r)* should directly influence each other. In comparison, of the 33 participants who chose the non-addition form, none suggested that initial speed (*p)* and acceleration (*r)* should be kept separate from each other. Eight (29%) conceptualized p and r as directly influencing each other. The remaining participants for both choices used guesswork as well as other reasoning strategies (e.g., making sure the unit for speed is correct). A Chi-square test of independence indicated a significant association between equation choice and participants’ verbal reasoning, χ²(1, N = 61) = 14.99, p < .001. Participants who chose equations with the addition form were significantly more likely to verbalize that the initial speed (p) and acceleration (r) should be kept separate than those who chose non-addition forms.

## Discussion

Our study examined how different presentation formats in a simulation facilitate symbolic thinking in physics. An Animated Simulation condition presented a physics simulation as visual objects in motion, and a Textual Simulation condition described simulation results in a structured verbal format. Participants then attempted to select a mathematical equation that expressed the relationships in the simulation. We found that participants in the Textual condition were twice as likely to select the correct equation than participants in the Animated condition. But given the low overall rates of selecting the correct equation, this difference was not significant. However, when broadening the outcome to include any equation that had the accurate symbolic form (an additive relationship between initial speed *(p)* and acceleration *(r)*), participants in the Textual condition were significantly more likely to select this form relative to both the Control condition and the Animation condition. While this suggests that the Textual condition facilitates the choice of correct symbolic form, the lack of significant differences in correctly identifying the precise target equation highlights the need for caution in interpreting these effects.

At the same time, these findings confirm our hypothesis regarding the benefits of verbal materials in facilitating symbolic thinking. From the perspective of dual-coding theory [10], the textual and animated simulations engaged different representational systems. While the Animated condition primarily engaged the visual system, the Textual condition relied more on the verbal system. Because both text and equations are processed symbolically and discretely in the verbal system, textual descriptions may have facilitated stronger associative links between the verbal presentation of motion (“the ball started with a speed of 20 and began slowing down…”) and the equation of motion (q = p + rt). This correspondence between the structure of text and that of equations may have helped participants distinguish the initial speed from the subsequent change due to acceleration – an understanding that is central to choosing the addition form. Additionally, variability in textual descriptions prevented participants from making superficial associations between the word “and” and the addition form. Use of this word was not a significant predictor of the choice of addition form.

Qualitative exploration of participants’ verbal responses also indicated that the textual format directed attention towards the relationship between initial speed and acceleration. Participants who selected the equations with the addition form were significantly more likely to verbalize that the initial speed (*p*) and acceleration (*r*) variables should be kept separate from each other. This affordance of text to direct attention to relevant parts of a mechanical system has been demonstrated in previous research [40], and our study suggests that this affordance of text also has the potential to improve symbolic thinking. At the same time, it is important to recognize that the current study examined a version of the textual format that was both structured and narrative-like. These features may have provided additional explanatory depth and scaffolding and contributed to the observed effects. It remains unclear whether a non-narrative, unstructured textual format (e.g., “Initial speed = 20. Acceleration occurs. Time progresses to t = 8.”) would produce similar benefits. Future research might carefully disentangle the distinct contributions of the form (e.g., discrete presentation) and the content structure (e.g., narrative vs. non-narrative) of textual materials in supporting symbolic thinking. In addition, a hybrid condition that combines animation with explanatory text would offer a valuable comparison.

Our examination of participants’ performance during the simulation activities rules out some additional explanations. For example, it does not appear that the Textual condition produced a more challenging learning environment that led to better encoding of the problem space (e.g., a “desirable difficulty,” Bjork & Bjork [42]). Participants in the Textual condition had similar rates of success solving the goal activities relative to participants in the animation condition. Similarly, it does not seem that participants in the Textual condition simply put in more effort relative to others; across the experimental conditions participants made a similar number of attempts on each of the six goal activities. This parity between the conditions in the number of attempts and the success rates of solving the simulation tasks also rules out the possibility that the Animated Simulation was more difficult to perceive. Studies of visual perception of acceleration find that people have a higher threshold for detecting acceleration than for simply detecting movement (e.g., Calderone & Kaiser [43]). Nevertheless, people can detect changes in speed with relatively short presentation times [44], and the fact that there was no difference in success rates or number of attempts required suggests that the visual discernibility of the movement patterns during the Animated Simulation presented no challenges for task completion.

However, one key difference in their simulation activity emerged; participants in the Textual condition were more likely to employ a Control-of-Variables (COV) strategy in which they manipulated only one slider at a time while keeping the others constant. The difference in learning strategies for both the conditions also highlights the importance of textual simulations. A higher proportion of COV strategy trials indicate that participants used the structured information in the Textual condition to vary parameters with a hypothesis-testing driven mindset. They tried achieving success by understanding the mechanism underlying the motion of the ball. Even though differences in the use of this strategy did not significantly predict their choice of the *addition* form on the outcome measure, our results nevertheless indicate how presentation format can influence problem solving strategies [45]. It is possible that the effectiveness of the textual format results from its use in an interactive-goal driven context. To clarify this issue, future research could include a non-interactive textual control condition that presents the same explanatory information without requiring active manipulation of variables. Such a comparison would help determine whether it is the explanatory text, or its combination with an interactive-goal driven context, that most effectively supports symbolic thinking.

Previous research has examined how referential connections between visual and verbal learning materials best support problem solving [12]. This has led to design principles emphasizing the importance of temporal contiguity (i.e., simultaneously presenting visual animations with verbal narrations) and spatial contiguity (i.e., presenting text descriptions next to visual graphics) to optimize learning [46]. Rather than focusing on these connections, our work examines the individual benefits for each of these systems in the context of symbolic thinking. Further, our results suggest that in certain contexts and for certain learning outcomes, simulation presented in a structured textual format alone may provide sufficient support for meaningful symbolic thinking. This demonstration has value because some researchers have advocated for the opposite – *reducing* non-perceptual elements in physics learning simulations (e.g., “the richer the perceptual experience, and therefore the mental perceptual simulation acquired, the better the student learning and understanding,” Black et al. [47]). Thus, even while visual and verbal presentation formats are not mutually exclusive in practice (ideally, a learning simulation would include both), the current study provides a useful demonstration that textual information, when presented in a structured and narrative-like format, can support a learners’ symbolic thinking. These findings have direct relevance for the design of instructional materials in real educational settings. Rather than positioning text and animation as competing modalities, future studies might integrate the intuitive advantages of animation with the precision offered by textual scaffolds. To our knowledge, no prior study has systematically combined interactive variable manipulation with dynamically generated, narrative-like textual feedback that explains the consequences of those manipulations. Most learning simulations represent changes in variables primarily through perceptual updates—such as animations or visual transformations—without accompanying linguistic scaffolds. Our study suggests that this pairing of interactivity with explanatory text may offer a powerful, yet underutilized, approach to supporting symbolic thinking. These findings may be especially relevant for novice learners in physics or other STEM domains in which learners lack knowledge of critical features that they ought to attend to (e.g., Miyatsu et al. [48]). Future research should test hypotheses such as whether: (1) combining animation with textual scaffolding yields better symbolic thinking than either format alone, (2) textual scaffolding is more beneficial for novice learners than for those with greater domain expertise, and (3) these effects persist or shift in the context of more complex mechanical systems involving multiple interacting variables or nonlinear dynamics. These hypotheses can inform not only future studies but also the development of simulation-based learning environments that are both perceptually rich and precise.

In terms of limitations, our study does not demonstrate whether these findings generalize to advanced physics learners. The sample primarily consisted of introductory-level psychology students with limited physics experience. We also don’t know whether these findings generalize to topics beyond acceleration, which employ more complex equations in physics. It is possible that for equations describing mechanical systems with multiple components, a textual format might be helpful in directing attention to relevant components since learning happening solely through animation would be harder. We also don’t know if these findings would generalize to equations that do not use the addition form. It is possible that the textual format might not be adept at describing relationships which are expressed using multiplicative forms or a combination of different symbolic forms.

Finally, it is important to understand that symbolic forms are a necessary but not sufficient marker of symbolic thinking. As discussed in the symbolic forms [7, p.504] literature, these knowledge elements capture the conceptual schema and symbolic template embedded in an equation. Identifying the correct form is important for reasoning meaningfully with the symbols. However, symbolic forms are not the only knowledge elements learners use during symbolic thinking. Participants’ verbal explanations indicated the use of other knowledge elements like units, arithmetic estimation, and algebraic reasoning. All these elements work together in improving a learner’s symbolic competence in physics [49]. Our study primarily focused on symbolic thinking from the perspective of symbolic forms. It is also important to note that symbolic thinking can not fully be assessed by identifying the correct equation or the correct symbolic form. A learner’s ability to apply this equation meaningfully across a variety of contexts is a key marker of symbolic competence. Future work should investigate meaningful connections between a learners’ ability to identify the correct symbolic form and performance on transfer tasks.

The ability to understand a phenomenon conceptually, and to express that conceptual understanding mathematically, is foundational to scientific thinking [5]. Overall, our study utilizes the dual-coding framework to examine how visual and verbal presentation formats constrain or enhance this ability, respectively, in a physics simulation context. In the last few decades, technological advances have introduced multiple presentation formats in physics learning environments, particularly using simulations [17]. These formats vary on a spectrum of concreteness, with some closely resembling our perceptual experiences in the world and others which are increasingly abstract and symbolic in nature. By directing attention to mechanistically relevant features across these learning formats, verbal materials can provide an appropriate scaffold and improve symbolic thinking in physics.
